# Exosomes in Human Immunodeficiency Virus Type I Pathogenesis: Threat or Opportunity?

**DOI:** 10.1155/2016/9852494

**Published:** 2016-01-04

**Authors:** Sin-Yeang Teow, Alif Che Nordin, Syed A. Ali, Alan Soo-Beng Khoo

**Affiliations:** ^1^Molecular Pathology Unit, Cancer Research Centre (CaRC), Institute for Medical Research (IMR), 50588 Kuala Lumpur, Malaysia; ^2^Faculty of Health Sciences, Universiti Teknologi MARA (UiTM), Bertam Campus, 13200 Kepala Batas, Pulau Pinang, Malaysia; ^3^Oncological and Radiological Sciences, Advanced Medical and Dental Institute, Universiti Sains Malaysia, 13200 Kepala Batas, Pulau Pinang, Malaysia

## Abstract

Nanometre-sized vesicles, also known as exosomes, are derived from endosomes of diverse cell types and present in multiple biological fluids. Depending on their cellular origins, the membrane-bound exosomes packed a variety of functional proteins and RNA species. These microvesicles are secreted into the extracellular space to facilitate intercellular communication. Collective findings demonstrated that exosomes from HIV-infected subjects share many commonalities with Human Immunodeficiency Virus Type I (HIV-1) particles in terms of proteomics and lipid profiles. These observations postulated that HIV-resembled exosomes may contribute to HIV pathogenesis. Interestingly, recent reports illustrated that exosomes from body fluids could inhibit HIV infection, which then bring up a new paradigm for HIV/AIDS therapy. Accumulative findings suggested that the cellular origin of exosomes may define their effects towards HIV-1. This review summarizes the two distinctive roles of exosomes in regulating HIV pathogenesis. We also highlighted several additional factors that govern the exosomal functions. Deeper understanding on how exosomes promote or abate HIV infection can significantly contribute to the development of new and potent antiviral therapeutic strategy and vaccine designs.

## 1. Introduction

The membrane-bound exosomes are present in a wide range of human fluids such as urine [1], plasma [2], saliva [3], ascites [4], breast milk [5], semen [6], bronchoalveolar lavage liquid [7], amniotic fluid [8], and cerebrospinal fluid [9]. These microvesicles are secreted from various types of immune cells such as dendritic cells (DCs) [10], macrophages [11], T cells [12], and B cells [13], as well as tumor cells from various cancers [14, 15]. Exosomes are mainly responsible for cell-cell communication processes such as cell proliferation [15], cell invasion [16], and immune and gene regulation [17, 18]. It is known that exosomes are derived from cellular endosomes, where the inward budding takes place on the endosomal multivesicular bodies (MVBs) to form the intraluminal vesicles (ILVs) [19]. The subsequent molecular mechanism then determines the fate of ILVs, entering the lysosomal degradation pathway or released extracellularly as exosomes upon fusion of MVB membrane with the plasma membrane [20].

Accumulative findings have demonstrated that exosomes highly resembled HIV particles in many aspects, from their physical properties to composition [21–24]. This has given rise to two models that explain these similarities [24]. First, the Trojan exosome hypothesis proposed that retroviruses are originated from exosomes following the evolution involving* gag* gene mutation [25]. This explained the ability of virus to exploit the preexisting exosome biogenesis pathway for viral dissemination and be able to infect cells in Env- and receptor-independent manner [26, 27]. The second model, however, is not in line with the evolutionary theory of the virus. Instead, the “crosstalk” or “hijacker” hypothesis suggested that the retroviruses have evolved to hijack the intercellular communication pathway of the host to promote HIV pathogenesis [28]. Although both models differ from each other, the similarity of the compositions (i.e., lipids, proteins, carbohydrates, and RNAs) between viral particles and exosomes suggests that exosomes may play an indispensable role in HIV pathogenesis.

Recently, several reports have demonstrated that exosomes contain internal cargoes that can inhibit HIV infection and replication [29–31]. These antiviral exosomes were mostly found in the body fluids such as semen and breast milk. However, the inhibitory action of exosomes is not well described compared to its viral infection enhancement effects. This may be due to the high abundance of HIV pathogenesis promoting molecules within the composition of exosomes, which may mask the existing antiviral effects, if any. By far, collective findings have shown that exosomes can either promote or inhibit HIV infection, with little understanding upon the critical factors and/or the exact mechanisms that determine the exosomal effects in viral infection. In general, the source (i.e., from different cell types and biological fluids) and the composition of exosomes may exert the decisive role in contribution to HIV/AIDS pathogenesis. More effort is required to thoroughly understand the exosomal function in HIV infection in order to benefit the development of new-era HIV/AIDS therapy and vaccine designs.

## 2. Morphological and Biological Properties of Exosomes and HIV Particles

Exosomes share several common structural and molecular properties with HIV. Physically, their size and density range from 50 to 150 nm in diameter [32] and 1.13 to 1.21 g/mL [33], respectively, and both are surrounded by a lipid bilayer. In addition to morphological similarities, they possess similar composition such as lipids (i.e., cholesterol and glycosphingolipids) [13], carbohydrates (i.e., high mannose and complex N-linked glycans) [34], proteins (i.e., tetraspanins, MHC molecules, actin, and TSG101) [35, 36], and RNA species [24]. Exosomes from HIV-infected cells are also enriched with viral proteins such as Nef [37] and viral RNAs [18, 38]. Due to these similarities, HIV-1 is believed to be generated by the same pathway of exosome biogenesis [24, 39]. Moreover, a substantial amount of the host component (e.g., MHC-II) can enter the viral particles [25]. This can be one of the mechanisms that is exploited by viruses to evade the host immune surveillance.

Despite sharing most of the biochemical features, HIV particles have a few principal differences in comparison with exosomes. First, HIV has more organized and uniform structures regardless of the type of infected cells while the structure of exosomal vesicles varies depending on the parental cell after the membrane budding [40]. Second, the exosomal contents are highly diverse from different sources while the biochemical content of HIV virions is steadily consistent. These differences allow the purification methods based on iodixanol density gradients and immunoaffinity isolation to efficiently harvest exosome-free HIV virions [40, 41].

## 3. Distinct Functions of Exosomes from Different Sources

The composition of exosomes derived from biological fluids is highly variable from one to the other, which suggest the composition may define the distinct effects of exosomes (either promoting or inhibiting viral pathogenesis). Cumulative evidence suggested that the exosomal effect on HIV mainly depends on their cellular origins [42]. In most cases but not all, exosomes derived from HIV-infected cells are more virulent and enhance infection, while exosomes from uninfected cells have protective properties. In this section, we discuss the exosomal functions and their effects on HIV pathogenesis based on their sources or origins (summarized in Table 1).

### 3.1. Blood/Plasma/Serum

Human blood is where the HIV virions reside and is the main biofluid responsible for HIV transmission. The blood also contains various types of cells of both HIV-susceptible and uninfected cells that secrete exosomes. It has been postulated that HIV hijacks the exosome biogenesis pathway which carries various viral proteins and RNAs for viral dissemination process [24]. A general review on how exosomes enhance the spread of various infections has been recently reported [23, 42]. Exosomes secreted from HIV-infected cells had been found to contain chemokine receptors, CCR5 and CXCR4, that were delivered to recipient cells to facilitate HIV establishment and spreading [43, 44]. Exosomes from HIV-1 infected macrophages had also been known to facilitate viral transfer to uninfected cells [45]. Exosomes that contain HIV Nef protein have multiple pathogenic effects such as induction of T-cell apoptosis [37], inhibition of RNA interference [46], and downmodulation of cell surface molecules (i.e., MHC-I and CD4) for immune evasion [47]. Nef protein also induces exosomal secretion [37], thereby contributing to HIV/AIDS pathogenesis. Unlike the transfer of CCR5 and/or CXCR4 which primarily direct HIV infection, Nef proteins promote HIV infection by activating the uninfected cells.

Other viral components that are usually found in exosomes are HIV Gag [39], viral mRNA/miRNA [18], and pathogen-associated RNAs such as HIV trans-activation response (TAR) RNA [48] that could enhance the viral infection and replication in the recipient cells. Various host surface molecules (CD45, CD86, and MHC-II) have also been exported from HIV-infected cells via exosomes to silence the immune response [49]. Additionally, exosomes derived from infected dendritic cells (DCs) have a profound enhancing effect on CD4+ T cell infection [50]. More recently, Nef-induced exosome-associated ADAM17 (ADAM metallopeptidase domain 17) had rendered resting CD4+ T cells permissive to HIV-1 infection [51], whereas ADAM17 along with TNF-*α* has been known to synergistically activate the latent HIV-1 in primary CD4+ T lymphocytes and macrophages [52]. HIV particles are known to incorporate various host cell components that enable the viruses to evade the immune system. The exchange of these components could be facilitated by the existing exosomes that are enriched with the host components. While extensive studies have been conducted on exosomes derived from HIV-infected cells, little is known of the role of exosomes derived from uninfected cells in viral pathogenesis. The increased exosomal secretion may have increased the susceptibility of uninfected cells to HIV; this however warrants future investigation.

Although exosomes are mainly enriched with viral components that promote HIV/AIDS pathogenesis, a few reports have shown that exosomes may potentially inhibit HIV infection. APOBEC3G, a host cellular protein, has been known to transfer from cell to cell through exosomes to protect the recipient cell from HIV infection [29, 53, 54]. APOBEC3G, the most prominent member of APOBEC3 (A3) proteins, is a cellular cytidine deaminase that restricts HIV replication by both DNA-editing and editing-independent activities [55, 56]. In order to function, the APOBEC3G must be incorporated into the virions [57]. The extensive role of APOBEC3G in antiviral immunity has been recently reviewed [58]. The restrictive effect is more prominent in* Vif*-deficient HIV-1 than the wild-type strain as the Vif proteins target and counteract APOBEC3G for polyubiquitination and degradation by the 26S proteasome [29]. APOBEC3G proteins have been detected in human mammary tissues and were packaged into milk-borne virions and subsequently restricted HIV-1 infectivity [53]. Besides, it has also been shown that APOBEC3G inhibited viral replication by blocking the function of HIV-1 reverse transcriptase [54].

Although T lymphocytes are the primary reservoir of HIV infection, exosomes released from T lymphocytes had been found to have inhibitory effects against HIV. When compared to CD4-depleted exosomes from CD4+ T cells, CD4-containing exosomes efficiently inhibited HIV-1 infection [59]. This may be due to the masking of HIV-1 envelope proteins by the exosomal CD4 that has subsequently blocked HIV infection. Similarly, exosomes secreted from CD8+ T cells were able to suppress HIV transcription within the infected cells [60]. Other components from exosomes that inhibit viral infections are interleukins [61, 62], interferon-alpha [63], interferon-beta [64], and tumor necrosis factor (TNF-*α*) [61, 65].

### 3.2. Breast Milk

While exosome's dual-functions were seen in the blood-derived exosomes, current findings on milk-derived exosomes are skewed towards their antiviral effects, probably due to its role in providing natural passive immunity for infants. Exosomes were found to contain several components such as Lewis X [66], bile lipase [67], antibodies [68], mucin 1 (MUC-1) [69], and oligosaccharides [70] that inhibit the DC-mediated HIV transmission to CD4+ T lymphocytes. More recently, the antiviral effect of exosomes has been shown to be specifically derived from the milk since no HIV-1 inhibition was seen in plasma-derived exosomes when experimented in parallel [31]. In this study, the milk exosomes bound to monocyte-derived dendritic cells (MDDCs) and inhibited HIV-1 infection of MDDCs and the subsequent viral transfer to CD4+ T cells. Cumulative work showed that milk exosomes have a strong inhibitory effect; these protective effects may be transferred to the uninfected cells of newborns via breastfeeding as part of the passive antiviral immunity. This may also be a reason why the HIV-1 transmission via breastfeeding is rare. More efforts are currently in progress to evaluate the potential of utilizing milk exosomes in the antiviral therapy [42].

### 3.3. Semen

HIV-1 infection is notoriously known to be transmitted through sexual intercourse. Surprisingly, the semen-derived exosomes mainly possess antiviral effects compared to mediating HIV infection. It has been shown that exosomes purified from healthy individuals inhibit the HIV-1 replication in various cell types by blocking the postentry viral RNA reverse transcription [30]. The same group has also shown that exosomes in human semen were able to restrict the HIV transmission* in vivo* in LP-BM5-infected mice model [71]. Another group has also reported that the mucin-containing exosomes were able to prevent the HIV-1 transfer from DCs to CD4+ T cells [72]. Similar to the milk exosomes, these exosomes may presumably be transferred from one to another to exert the antiviral or protective effects. Yet, this seems to be inefficient as the number of HIV cases due to sexual transmission increases every year. Although several reports have highlighted the protective role of semen-derived exosomes, the exact mechanism that is involved is still unknown. This is important in order to control or prevent the HIV spreading, particularly through unprotected sexual intercourse.

### 3.4. Urine, Saliva, Ascites, and Other Biological Fluids

While the anti-HIV action of exosomes has been shown in human blood, semen, and breast milk, its antiviral potential in other sources such as saliva and ascites is yet to be determined. Since the composition of exosomes is heterogenous depending on the origins, it is of crucial importance to understand the factors that may eventually lead to its viral or antiviral effects. More efforts need to be done to reveal the activity of exosomes and the mechanisms in these biofluids as they may serve as an important source for antiviral therapies.

## 4. Decisive Factors for Exosome Functions in HIV Infection

It is interesting that the exosome nanovesicles packed with a very complex composition displayed two contradictory functions towards HIV infection (Table 1). However, little is known about the criteria that drive the ultimate role of exosomes in HIV/AIDS pathogenesis. Cumulative findings have demonstrated that the function of exosomes is mainly directed by their cellular origin and composition. Exosomes derived from HIV-infected T cells, monocyte/macrophage, and dendritic cells contain several components that abate viral infection [21, 24, 45]. These immune cells may release immune-regulatory factors that possess antiviral property such as interferon-alpha (IFN-*α*) [63], interferon-beta (IFN-*β*) [64], tumor necrosis factor-alpha (TNF-*α*) [65, 66], interleukins (ILs) [66, 67], and APOBEG3G [29, 53, 54], which are exported by exosomes from the cells. However, this antiviral implication might be masked due to the lytic replication of HIV that resulted in the pathogenic effects. To note, some of the viral molecules such as Nef and viral TAR RNA have been detected in exosomes which may further enhance the infection [37, 48]. Similarly, the antiviral activity of exosomes has been found in biological fluids (i.e., semen and breast milk) that are rich in immunological molecules [30, 31]. This indicates that the function of exosomes partly depends on the cellular origin. However, the exact components and their underlying mechanism that contributes to the antiviral action are left to be discovered.

In addition to the cellular origin of exosomes, the target or recipient cells in which exosomes are delivered also play a pivotal role in viral pathogenesis. Exosomes are known to transport from HIV-infected CD4+ T cells among themselves [51] as well as to other recipients cells such as dendritic cells and macrophages [45, 73]. The dendritic cells were also known to capture exosomes from the exosome-producer cells or infected CD4+ T cells and export to HIV-susceptible cells for the infection. These processes were termed as* trans*-infection and* trans*-dissemination, respectively [73, 74]. During the process, viral proteins such as chemokine receptors and Nef may be delivered to the recipient cells, thereby enhancing HIV infection and replication. Indeed, the ultimate functions of exosomes in the HIV-infected individuals largely rely on the composition of the nanosized exosomes. When both surface molecules and internal cargoes of exosome have net pathogenic effects, there is more likelihood that exosomes would contribute to viral pathogenesis and* vice versa*. The components of exosomes may also alter the intracellular signalling pathways [75, 76], thereby affecting HIV-1 infection.

It is well-known that the content of exosomes largely varies depending on their origins. However, it is intriguing that exosomes derived from the same source (i.e., blood-derived exosomes) have opposing effects in HIV pathogenesis. While individual components within the exosomes appear to have opposing effects on HIV pathogenesis, the net effect of the exosomes may depend on the relative strength of the effects. In addition to the net pathogenic effect of exosomal contents, it is noteworthy that variation in testing conditions such as culture conditions, exosome or virions preparation, cell infection status, and exosomal transfer or delivery status may also play role in determining the outcome of exosomes in viral infection. For example, the profile of exosomes generated* in vitro *from cultured cells may differ drastically from exosomes that are freshly harvested from biofluids. The variation of exosomes derived from tumor cells cultured* in vitro *and* in vivo *had been previously reported [77]. In this case, the experiments utilizing exosomes prepared* in vitro *may not represent the* in vivo* exosomal effects on viral infection. Second, the different methods used in exosome or virions preparation may also complicate the data interpretation. Exosomes prepared* in vitro* might be contaminated with HIV virions during the purification, thereby affecting the outcome of exosomes on viral pathogenesis. Third, the efficiency of exosomal transfer from the exosome producer cells to the recipient cells may also play a key role in viral pathogenesis. For instance, exosomes derived from cultured cells* in vitro *may not be as potent in the exchange of exosomal contents (i.e., virulence factors and/or antiviral components) as compared with* in vivo *transfer [42]. Moreover, the level of exosome secretion is dependent on the HIV infection status as Nef proteins mediate the exosome release [37]. All of these factors have to be taken into consideration by researchers before concluding the study outcome.

## 5. Development of a New-Era HIV Therapeutic Strategy

Cumulative findings demonstrate that the cell-encoded exosome pathways enhance HIV infection, supported by the Trojan exosome hypotheses [25] and the envelope protein- and receptor-independent viral dissemination without involving fusion events [26]. On the other hand, exosomes derived from the breast milk and semen exhibit modest antiviral effects [30, 31]. Both of these contradictory functions of exosomes pose a major impact to the existing strategy of anti-HIV drug development and urge the discovery of new-era therapeutics. For instance, the Env-targeting therapeutics may contribute to a low extent or none at all to the exosome-mediated infection that can take place without Env-receptor fusion. Hence, a more comprehensive strategy is needed to control the infection. The exosomal pathway in which HIV was hijacked for the viral dissemination can be targeted by newly designed inhibitors to target exosome biogenesis and exosome uptake in both Env-dependent and independent infections. Potential exosomal targets are cellular enzymes (RNases, proteases, and lipase), cytoplasmic proteins (TSG101, cyclophilins, MHC-II, and tetraspanins), and HIV-related proteins (e.g., Nef, CCR5, and CXCR4) [21, 23]. However, the specificity of the therapy must be monitored as the host-derived exosomes may trigger deleterious side effects. Several reports have shown that exosomal inhibitors have reduced the overall Env-dependent infection [30, 31] and these suggest the applicability of inclusion of exosome inhibitors in the adjunctive HIV therapy. Exosomes that exhibit antiviral activity can be purified from the particular sources and developed into a potential therapy. Indeed, more promising action is envisioned when the exact components and mechanisms are revealed. It would also be interesting to determine whether the relative amount of HIV promoting versus HIV inhibiting exosomes could affect disease progression and thereby serve as potential biomarkers for prognosis. While this review focuses on the role of exosomes in HIV infection, these nanovesicles have also been shown to contribute to pathogenesis of other viral classes such as Hepatitis B and C virus [21, 78] and Herpes Simplex virus (HSV) [22]. Continuous efforts must be made to enhance the understanding of the exosomal function in these viral infections and their potential use in the development of antiviral therapies.

## 6. Conclusions

To summarize, the host-derived exosomes enhance HIV infection by several routes, including intercellular dissemination of viral components and immune evasion whereas only a limited number of reports have shown the antiviral function of exosomes. Numerous factors, such as the cellular origins, recipient cells, and the intracellular signalling that are affected by exosomes, are currently thought to contribute to the final outcome of exosome in the infection. Variation in exosome preparation and testing conditions may significantly affect the outcome of exosomes. Compared to other fields, the exosomal functions in HIV and other types of viral infections are apparently underexplored. More research is anticipated to improve the understanding of the association between exosomes and viral infections in order to reveal the potential of exosomes in the development of anti-HIV therapy.

## Figures and Tables

**Table 1:** Dual effects of body fluids-derived exosomes against HIV infection.

Viral/antiviral effect	Exosomal source	Active component	Reference
Promote HIV infection	Blood/plasma/serum	CCR5 and CXCR4	[43, 44]
		Nef	[37, 46, 47]
		Gag	[39]
		Viral mRNA/miRNA	[18]
		TAR RNA	[48]
		CD45, CD86, and MHC-II	[49]
		ADAM17, TNF-*α*	[51, 52]
		Undefined (from DCs)	[50]

Inhibit HIV infection	Blood/plasma/serum	APOBEC3G	[29, 53, 54]
		CD4	[59]
		Interferon-alpha (IFN-*α*)	[63]
		Interferon-beta (IFN-*β*)	[64]
		Tumor necrosis factor (TNF-*α*)	[61, 65]
		Interleukins	[61, 62]
		Undefined (from CD8+ T cells)	[60]
	Breast milk	Lewis X	[66]
		Bile lipase	[67]
		IgA and IgG antibodies	[68]
		Mucin 1 (MUC-1)	[69]
		Oligosaccharides	[70]
		Undefined	[31]
	Semen	Mucin 6	[72]
		Undefined	[30, 71]

Promote/inhibit HIV infection (unexplored)	Urine	Unexplored	Unexplored
	Saliva		
	Ascites		
	Bronchoalveolar lavage liquid (BAL)		
	Amniotic fluid		
	Cerebrospinal fluid		
	Vaginal fluid		

## Abbreviations

ADAM17: ADAM metallopeptidase domain 17
AIDS: Acquired immunodeficiency syndrome
CD: Cluster of differentiation
DCs: Dendritic cells
Env: Envelope protein
HIV: Human immunodeficiency virus
ILVs: Intraluminal vesicles
MDDCs: Monocyte-derived dendritic cells
MHC-II: Major histocompatibility complex class II
MUC: Mucin
MVBs: Multivesicular bodies
TAR: trans-Activation response.
